# Toward the Positive Consequences of Teacher-Student Rapport for Students' Academic Engagement in the Practical Instruction Classrooms

**DOI:** 10.3389/fpsyg.2021.759785

**Published:** 2021-10-05

**Authors:** Xiuqin Zhou

**Affiliations:** College Students' Mental Health, Education Center Shandong, Management University, Jinan, China

**Keywords:** teacher-student rapport, student academic engagement, practical instruction classrooms, positive consequences, interpersonal communication behavior

## Abstract

Due to the fact that teacher-student rapport may favorably influence students' academic behaviors, several scholars have empirically studied the impact of this interpersonal communication behavior on a range of student-related variables. Notwithstanding, academic engagement as another student-related variable has received less empirical attention. Further, no review study has been carried out to illustrate the beneficial outcomes of teacher-student rapport for students' involvement. The current study, hence, aims to fill these gaps by explaining the construct of teacher-student-rapport and its positive consequences for students' academic engagement in the practical instruction classrooms. Drawing on the available evidence, the positive impact of teacher-student rapport on students' academic engagement was illuminated. The significant implications of the finding are also discussed.

## Introduction

In classroom interactions, teachers and students may influence each other either positively or negatively (Luo et al., 2020). A negative teacher-student relationship may lead to stress, anxiety, and aggression in students (Hashemi, 2011; Alnuzaili and Uddin, 2020). Accordingly, creating a positive relationship with pupils is among the top priorities of teachers in any educational setting, especially in the practical instruction classrooms. A positive and favorable relationship between teachers and students is called teacher-student rapport (Frisby and Martin, 2010). Reyes and Von Anthony (2020) defined this construct as “a harmonious teacher–student relationship which encompasses enjoyment, connection, respect, and mutual trust” (p. 2). As put forward by Wilson et al. (2010), to establish rapport in classrooms, teachers should pay attention to students' interests, value their beliefs and ideas, and allow them to freely express their feelings toward instruction.

In addition, having a sense of humor and providing continuous feedback are also enumerated as the approaches through which instructors can create a close relationship with their pupils (Frisby et al., 2017). To illuminate the significance of teacher-student rapport, Ibarra (2014) stated that the strong rapport between teachers and students can contribute to desirable academic behaviors. In this regard, Nathan (2018) postulated that those instructors who are able to build a harmonious relationship with their pupils can effectively improve students' sense of accomplishment, which contributes to their increased autonomy.

Additionally, Xie and Derakhshan (2021) also illustrated that positive teacher interpersonal behaviors such as teacher-student rapport can positively and dramatically influence student learning outcomes. Given the importance of teacher-student rapport in academic contexts, several studies have explored the positive outcomes of this factor for students' motivation (e.g., Opdenakker et al., 2012; Koca, 2016; Frisby et al., 2017; Henry and Thorsen, 2018; Zheng et al., 2021), learning achievement (e.g., Yunus et al., 2011; Hughes et al., 2012; Wubbels et al., 2016), and academic success (e.g., Camp, 2011; Estepp and Roberts, 2013; Jimerson and Haddock, 2015; Lammers et al., 2017). Yet, the desirable consequences of teacher-student rapport for other student-related variables such as academic engagement have received less attention (e.g., Estepp and Roberts, 2015; Geng et al., 2020).

In a general sense, student academic engagement refers to “the quality of students' participation or connection with the educational endeavor and hence with activities, values, individuals, aims, and place that comprise it” (Skinner et al., 2009, p. 496). When it comes to the practical instruction classroom contexts, student academic engagement pertains to the amount of effort that learners dedicate to learn a new language (Hiver et al., 2021). Barkatsas et al. (2009) stated that students' academic engagement can lead to increased achievement, enhanced retention, and academic success. That is, those students who exert more effort in doing classroom activities are more likely to acquire course content. Thus, exploring factors that may positively contribute to students' academic engagement seems essential.

In line with this necessity, some empirical studies (e.g., Ghelichli et al., 2020; Derakhshan, 2021; Jiang and Zhang, 2021) have examined the desirable outcomes of various personal and interpersonal factors for student academic engagement. However, as previously mentioned, the favorable effects of teacher-student rapport as an important interpersonal factor on students' academic engagement have been less investigated. Additionally, no review study has been carried out to illustrate the beneficial consequences of teacher-student rapport for students' academic engagement. To fill this gap, the present review study attempts to explain the positive consequences of teacher-student rapport for student academic engagement in the practical instruction classrooms.

### Teacher-Student Rapport

As a positive interpersonal factor, teacher-student rapport is conceptualized as “an emotional connection between teachers and their pupils based on understanding, caring, and mutual respect” (Lammers and Byrd, 2019, p. 128). Teacher-student rapport is a close bond between instructors and learners that enables them to work jointly in classroom contexts (Culpeper and Kan, 2020). As put forward by Weimer (2010), valuing students' ideas and viewpoints is a key to build a strong rapport with them. In this regard, Estepp and Roberts (2015) postulated that demonstrating concern for students' welfare is also essential for establishing a positive relationship with them. Taken together, those teachers who respect students' ideas and pay attention to their well-being are able to develop a strong connection with their pupils. Building rapport in classrooms is of high importance, mostly due to the fact that having close relationships with students motivates them to collaborate with instructors in order to attain their mutual objectives (Frisby et al., 2016).

### Student Academic Engagement

Student academic engagement refers to “the quality of the effort students themselves devote to educationally purposeful activities that contribute directly to the desired outcomes” (Hu and Kuh, 2002, p. 557). As a complex construct, student academic engagement comprises three components of “*Behavioral Engagement*,” “*Affective Engagement*,” and “*Cognitive Engagement*” (Jimerson et al., 2003). As the first sub-construct, behavioral engagement relates to students' active participation/involvement in academic tasks and activities. The second sub-construct of student academic engagement, known as affective engagement, refers to students' inner feelings regarding the instructional-learning context, peers, and instructors. Cognitive engagement, as the last component, pertains to the positive perceptions and attitudes of pupils toward their instructors, classmates, and the learning context (Alrashidi et al., 2016).

### The Positive Consequences of Teacher-Student Rapport for Students' Academic Engagement in the Practical Instruction Classrooms

To explain the positive consequences of teacher-student rapport, Ibarra (2014) stated that establishing friendly relations with pupils enables teachers to enhance students' willingness to engage in the learning process. In this regard, Pedler et al. (2020) also submitted that having positive relationships with teachers encourages students to enthusiastically participate in classroom tasks. Further, Xie and Derakhshan (2021) elucidated that positive interpersonal behaviors (e.g., confirmation, clarity, stroke, rapport, etc.) that teachers employ in instructional-learning contexts can remarkably promote students' learning engagement. Similarly, by relying on the basic assumptions of the positive psychology movement, Budzinska and Majchrzak (2021) suggested that students' academic behaviors such as engagement can be considerably enhanced in a positive learning atmosphere. To them, teachers can provide such pleasant atmosphere by developing a close and harmonious relationship with their pupils.

## Empirical Studies

Owing to the fact that teacher-student rapport can contribute to desirable academic behaviors (Ibarra, 2014; Wang et al., 2021), a large number of studies have probed into the positive consequences of this construct. Nevertheless, the beneficial effects of this positive interpersonal behavior on students' engagement have remained elusive. That is, compared to other academic behaviors, student academic engagement has received less attention (e.g., Estepp and Roberts, 2015; Geng et al., 2020; Snijders et al., 2020; Wanders et al., 2020). Estepp and Roberts (2015), for instance, examined the impact of teacher-student rapport on students' engagement. To do so, 306 university students were selected from different countries. To elicit participants' viewpoints, they were invited to respond to two close-ended questionnaires, namely *Professor-Student Rapport Scale (PSRS)* and *Student Academic Engagement Questionnaire* (SAEQ). Analyzing respondents' answers, they found that teacher-student rapport is a strong predictor of students' academic engagement. By the same token, Geng et al. (2020) also attempted to scrutinize the positive effects of positive teacher-student relationships on Chinese students' learning engagement. To this aim, 628 Chinese students took part in this study. They were asked to complete two reliable questionnaires. Inspecting the correlations of the utilized questionnaires, the researchers reported that there was a positive connection between positive teacher-student relationships and Chinese students' academic engagement. In their study, Wanders et al. (2020) also studied the probable association between teacher-student connection and students' involvement. In doing so, 4,128 students were opted from different schools in the Netherlands. The analysis of students' perceptions revealed a strong association between positive teacher-student relationships and student involvement.

## Conclusion and Pedagogical Implications

So far, different conceptualizations of teacher-student rapport and student academic engagement, their underlying dimensions, and their interrelationships were thoroughly explained. The previous studies conducted on these variables were also summarized to illuminate the positive consequences of teacher-student rapport for students' academic engagement. Drawing on the existing evidence, one can infer that favorable relationship between teachers and students can desirably influence students' academic engagement. An important implication of this is that those people who are in charge of training pre-service teachers should teach them how to establish pleasant and friendly relationships with students. It is due to the fact that inexperienced and novice teachers typically do not know how to establish a strong rapport with their pupils (Farhah et al., 2021). Another important implication that emerges from the finding of this study is related to both pre and in-service teachers. Since teacher-student rapport plays a pivotal role in fostering student academic engagement (Budzinska and Majchrzak, 2021; Wang et al., 2021; Xie and Derakhshan, 2021), teachers in any educational context should employ appropriate interpersonal behaviors to create a close relationship with students.

## Author Contributions

XZ confirmed being an independent author and submitted to this special issue.

## Conflict of Interest

The author declares that the research was conducted in the absence of any commercial or financial relationships that could be construed as a potential conflict of interest.

## Publisher's Note

All claims expressed in this article are solely those of the authors and do not necessarily represent those of their affiliated organizations, or those of the publisher, the editors and the reviewers. Any product that may be evaluated in this article, or claim that may be made by its manufacturer, is not guaranteed or endorsed by the publisher.
